# Bimodal Expansion of the Lymphatic Vessels Is Regulated by the Sequential Expression of IL-7 and Lymphotoxin α_1_β_2_ in Newly Formed Tertiary Lymphoid Structures

**DOI:** 10.4049/jimmunol.1500686

**Published:** 2016-07-29

**Authors:** Saba Nayar, Joana Campos, Ming May Chung, Leyre Navarro-Núñez, Menka Chachlani, Nathalie Steinthal, David H. Gardner, Philip Rankin, Thomas Cloake, Jorge H. Caamaño, Helen M. McGettrick, Steve P. Watson, Sanjiv Luther, Christopher D. Buckley, Francesca Barone

**Affiliations:** * Rheumatology Research Group, Centre for Translational Inflammation Research, Institute of Inflammation and Ageing, University of Birmingham Research Laboratories, Queen Elizabeth Hospital, Birmingham B15 2WD, United Kingdom;; † Cardiovascular Sciences, College of Medical and Dental Sciences, University of Birmingham, Birmingham B15 2TT, United Kingdom;; ‡ Medical Research Council Centre for Immune Regulation, College of Medical and Dental Sciences, University of Birmingham, Birmingham B15 2TT, United Kingdom; and; § Department of Biochemistry, University of Lausanne, 1066 Epalinges, Switzerland

## Abstract

Lymphangiogenesis associated with tertiary lymphoid structure (TLS) has been reported in numerous studies. However, the kinetics and dynamic changes occurring to the lymphatic vascular network during TLS development have not been studied. Using a viral-induced, resolving model of TLS formation in the salivary glands of adult mice we demonstrate that the expansion of the lymphatic vascular network is tightly regulated. Lymphatic vessel expansion occurs in two distinct phases. The first wave of expansion is dependent on IL-7. The second phase, responsible for leukocyte exit from the glands, is regulated by lymphotoxin (LT)βR signaling. These findings, while highlighting the tight regulation of the lymphatic response to inflammation, suggest that targeting the LTα_1_β_2_/LTβR pathway in TLS-associated pathologies might impair a natural proresolving mechanism for lymphocyte exit from the tissues and account for the failure of therapeutic strategies that target these molecules in diseases such as rheumatoid arthritis.

## Introduction

During inflammation or following immunization critical changes occur to the nonhematopoietic stromal component (fibroblast subsets, blood, and lymphatic endothelial cells) of the target tissue. Lymph node swelling occurs as a result of active stromal cell proliferation, accumulation of follicular dendritic cells, and the expansion and stretching of fibroblastic reticular cells, owing to mechanical changes that occur to the fibroblastic reticular cell cytoplasm (1). Similarly, changes in the vascular system occur upon immunization that allow a dramatic expansion of the lymphatic and vascular network that enables maximal cellular interaction and increases cellular output from the lymph node (2). The enlargement of the pre-existing lymphatic network is achieved by de novo formation of lymphatic vessels, also known as lymphangiogenesis (3–7). The cytokine IL-7 produced both by fibroblastic reticular cells and by lymphatic endothelial cells has been shown to contribute to this phenomenon during lymph node remodeling in a paracrine and autocrine manner (8, 9). Lymphotoxin (LT)α_1_β_2_/LTβR signaling is thought to contribute to the homeostatic regulation of the lymphatic vessels in secondary lymphoid organs (SLOs) (10). Interestingly, LTα_1_β_2_ signaling is also responsible for the formation and maintenance of fibroblast network, which in turn produces cytokines critical to preserve vascular integrity, such as vascular endothelial growth factor (VEGF)-A and -C (11, 12). We have recently shown how defects in lymphatic vessel formation in the lymph node anlagen profoundly impair development and function of these organs (13). Similarly, in adult life, interruption of lymphatic vessels is known to impair lymph node homeostasis (14), thus highlighting the reciprocal relationships that take place between vascular cells, the lymphatic system, and lymphoid fibroblastic cells in SLOs.

Tertiary lymphoid structures (TLS) are ectopic accumulations of lymphoid cells within peripheral tissue that share many cellular compartments, spatial organization, vasculature, chemokines, and function with SLOs. TLS form preferentially at mucosal sites in response to chronic antigenic challenge during infections or autoimmune diseases (i.e., in the salivary glands of patients with Sjögren’s syndrome or in the thyroid glands of patients with Hashimoto’s disease) (15–18). We and others have described the formation of activated stromal cell networks within TLS with the concomitant expression of lymphoid chemokines and cytokines (such as LTα_1_β_2_) that regulate lymphocyte clustering and organization (16, 19). TLS formation recapitulates some aspects of embryological SLO development. Moreover, Rorγt^+^ lymphoid tissue inducer (LTi) cells and activated stromal cells have been identified at these sites (16, 20–25). Although mechanisms leading to lymphangiogenesis in lymph nodes are relatively well understood, there is still limited information regarding the signals that regulate inflammatory lymphangiogenesis within TLS. Using a recently described, inducible model of TLS formation (26), we have dissected the expansion of the lymphatic vascular network within ectopic lymphoid organs that form in the salivary glands. We have observed expansion of the lymphatic endothelial cell (LEC) compartment and an increase in number of lymphatic vessels. This expansion, synchronous with the development of the inflammatory aggregates, results in progressive vascular splitting and is dependent on the presence of IL-7, LTα_1_β_2_, and infiltrating lymphocytes, in a similar manner to what is observed in SLOs. In our resolving model, this enlarged lymphatic system sustains lymphocyte egress from the tissue, suggesting that in TLS-associated diseases targeting the LT pathway might be counterproductive for the resolution of the lymphoid cell aggregates.

## Materials and Methods

### Mice and salivary gland cannulation

C57BL/6 mice were from Harlan Laboratories. *Ltßr^−/−^* mice, R*orc^−/−^* mice, *Rag2^−/−^* mice (on *boyJ* background), and *boyJ* mice were bred in the Biomedical Service Unit at the University of Birmingham. All mice were maintained under specific pathogen-free conditions in the Biomedical Service Unit at the University of Birmingham according to Home Office and local Ethics Committee regulations. Under ketamine/domitor anesthesia, the submandibular glands of female C57BL/6, *boyJ*, and knockout mice (8–12 wk old) were intraductally cannulated with 10^8^–10^9^ PFU of luciferase-encoding replication-defective adenovirus (Adv5), as previously described (15). Animals were recovered from anesthesia. Mice were culled by terminal anesthesia at days 2, 5, 8, 15, 23, or 26 after cannulation and salivary glands were harvested.

### In vivo blocking with anti–IL-7Rα

Rat anti-mouse anti–IL-7Rα Ab was used as described (27). Starting at day 0 postcannulation (p.c.), mice were administered a dose of 100 μg of Ab via i.p. injection followed by daily injections for 4 d.

### In vivo treatmet of *LtβR^−/−^* with recombinant VEGF-C

Recombinant VEGF-C (Abcam) was administered in the salivary glands of *Ltßr^−/−^* mice at the dose of 2 μg/gland at day 6 p.c., and mice were sacrificed at day 8 and glands were analyzed.

### Histology and immunofluorescence

Salivary glands from virus- or control vehicle-cannulated mice were harvested and snap frozen in OCT over liquid nitrogen. Six-micrometer-thick frozen sections were cut, left to dry overnight at room temperature, and stored next day in −80°C until use. For immunofluorescence analysis, slides were allowed to come to room temperature and then fixed for 20 min in ice-cold acetone, left to dry, and then hydrated in PBS. For immunofluorescence staining, all dilutions of reagents and Abs were made in PBS with 1% BSA. First, to block endogenous biotin, sections were treated with 0.05% avidin and 0.005% biotin for 15 min each and washed for 5 min with PBS in between the two incubations, followed by blocking with 10% horse serum for 10 min. Slides were then incubated for 60 min with cocktails containing the following primary Abs in PBS (1% BSA): gp38/podoplanin clone 8.1.1, CD4 Alexa Fluor 647, or CD4 Pacific Blue clone RM4-5 (from BD Pharmingen), CD31-biotin or CD31-FITC clone 390, CD19 Alexa Fluor 647 clone eBio1D3, CD3e-biotin clone ebio500A2, and retinoic acid–related orphan receptor (ROR)γt clone AFKJS-9 (all from eBioscience), and CCL21 (goat polyclonal). CD31 FITC-conjugated Abs were detected using rabbit anti-FITC (Sigma-Aldrich) and then goat anti-rabbit IgG-FITC (Jackson ImmunoResearch Laboratories, West Grove, PA). CCL21 Abs were detected using donkey anti-goat FITC (Jackson ImmunoResearch Laboratories) and then rabbit anti-FITC (Sigma-Aldrich), followed by goat anti-rabbit IgG-FITC (Jackson ImmunoResearch Laboratories). RORγt was detected with goat anti-rat FITC (SouthernBiotech) and then rabbit anti-FITC (Sigma-Aldrich), followed by goat anti-rabbit IgG-FITC (Jackson ImmunoResearch Laboratories). gp38/podoplanin was detected using goat anti-hamster biotin (Cambridge Bioscience, Cambridge, U.K.). Biotinylated Abs were detected using streptavidin–Alexa Fluor 555 or 488 (Molecular Probes). Hoescht (Molecular Probes) was used for nuclear stain. All secondary Abs were incubated for 30 min. Slides were mounted with ProLong Gold antifade reagent (Invitrogen Life Technologies).

Images were acquired on a Zeiss LSM 510 laser scanning confocal head with a Zeiss Axio imager Z1 microscope. Digital images were recorded in four separately scanned channels with no overlap in detection of emissions from the respective fluorochromes. Confocal micrographs were stored as digital arrays of 2048 × 2048 pixels with 8-bit sensitivity; detectors were routinely set so that intensities in each channel spanned the 0–255 scale optimally. The LSM 510 image examiner software was used to process these images.

### Lymphatic quantitation

To investigate the dynamics of lymphatic vessels during different phases of the inflammatory process in inflamed salivary glands, we stained for the lymphatics using LYVE-1 Ab (Abcam) and imaged the whole tissue section using the Leica DM6000 (as mentioned above). Using ImageJ software, we drew a region around the lymphatic vessels and estimated both the area covered by the lymphatic vessels and the total tissue area. These data were then used to calculate the size of lymphatic vessels as relative area covered by lymphatic vessels (percentage). We also counted the lymphatics in each tissue section to ascertain the number of lymphatics per area of tissue. Analysis was performed by two blinded observers.

### Isolation of stromal cells

Harvested salivary glands from virus- or vehicle control-cannulated mice were cut into small pieces and digested for 40 min at 37°C with gentle stirring in 1.5 ml RPMI 1640 medium containing collagenase D (3.7 mg/ml; from Roche), DNAse I (30 μg/ml; from Sigma-Aldrich), and 2% (v/v) FCS. The suspension was gently pipetted at 15-min intervals to break up aggregates. The remaining fragments were further digested for 20 min at 37°C with medium containing collagenase dispase (3.7 mg/ml) and DNAse I (30 μg/ml). The suspension was then gently pipetted to break up remaining aggregates until no visible fragments remained. During the final pipetting, EDTA was added to a final concentration of 5 mM to further reduce cell aggregates. Cells were then passed through a 70-μm mesh, washed twice, and were resuspended in RPMI 1640 medium containing 10% (v/v) FCS.

### Flow cytometry

Single-cell suspensions were stained for 30 min in PBS (with 0.5% BSA and 2 mM EDTA) with cocktails of the following Abs: CD31-FITC clone 390, gp38-PE clone 8.1.1, CD45 PerCP/Cy5.5 clone 30-F11 (from eBioscience), epithelial cell adhesion molecule (EPCAM) PE/Cy7 clone G8.8 (from BioLegend), Ki67–Alexa Fluor 647, and BrDU–Alexa Fluor 647 (BD Pharmingen). Afterwards cells were washed twice, resuspended, and then analyzed using a CyAn ADP (Dako) with forward/side scatter gates set to exclude nonviable cells. Data were analyzed with FlowJo software (Tree Star).

### In vitro tube formation assay

The tube formation assay was performed on 12-well plates coated with 100 μl of Matrigel (BD Biosciences, Oxford, U.K.) as previously described with modifications (1). After polymerization of Matrigel at 37°C for 30 min, human LECs (1.5 × 10^5^ cells/well) resuspended in 2 ml of MV2 growth medium (PromoCell, Heidelberg, Germany) were seeded to each well and incubated at 37°C, 5% CO_2_ for 1 h. The medium was then changed and treatments were added to the cells. FCS (20%) was used as a positive control. Recombinant LTα_1_β_2_ (R&D Systems Europe, Abingdon, U.K.) was used at 2 μg/ml.

The effect of the stated treatments on human LEC network formation was evaluated 5 h after their addition. Images were digitally captured using a Zeiss 0.16 numerical aperture Plan-Neofluar ×5 Ph1 lens on a Zeiss Axiovert 200 inverted high-end microscope (Zeiss, Welwyn Garden City, U.K.) and a Hamamatsu Orca 285 cooled digital camera using SlideBook software (Intelligent Imaging Innovations). Analysis of cellular networks was performed using Gilles Carpentier’s angiogenesis analyzer for ImageJ (available online at: http://image.bio.methods.free.fr/ImageJ/?Angiogenesis-Analyzer-for-ImageJ) by quantifying total branching length, number of junctions, and number of meshes in five different images per well. Comparisons between nontreated, FCS-supplemented, and LTα_1_β_2_-treated samples were carried out using a one-way ANOVA test followed by a Bonferroni posttest. Results shown are mean ± SD from three independent experiments.

### RNA isolation and quantitative PCR

Total RNA was isolated from salivary glands with an RNeasy mini kit (Qiagen) and the RNA was then reverse transcribed using a high-capacity reverse transcription cDNA synthesis kit (Applied Biosystems) according to the manufacturer’s specifications. Reverse transcription was carried out on Techne 312 thermal cycler PCR machine. Quantitative RT-PCR (Applied Biosystems) was performed on cDNA samples for LTβ and VEGF-C mRNA expression. β-Actin was used as an endogenous control. The primers and probes used were from Applied Biosystems. The quantitative real-time PCR was run in duplicates on a 384-well PCR plate (Applied Biosystems) and detected using an ABI Prism 7900HT instrument. Results were analyzed with the Applied Biosystem’s SDS software (SDS 2.3). We used the mean of two technical replicates (C_t_ values) to calculate the ΔC_t_ value. The C_t_ of the β-actin was subtracted from the target gene C_t_ value and the relative amount was calculated as 2^−ΔC^_t_. The relative quantity (RQ) expression values were calculated as 2^−ΔΔC^_t_, where ΔΔC_t_ is the difference between the ΔC_t_ values of cannulated salivary glands and the ΔC_t_ of noncannulated salivary glands. C_t_ values >34 were not accepted, nor were technical replicates with more than two cycle differences between them.

### Statistics analysis

Statistical analysis was determined for all analyses in figures (except Fig. 6) with a Student *t* test. Statistical significance in Fig. 6 was determined by a one-way ANOVA.

## Results

### Bimodal expansion of the lymphatic vascular network during TLS development

To dissect the dynamics of lymphatic vessel expansion in the context of ectopic lymphoneogenesis, we used a model of TLS formation in the salivary glands, where the single administration of a replication-deficient adenovirus via retrograde cannulation of submandibular glands is sufficient to induce focal aggregate formation as observed in human Sjögren’s syndrome (28). This model is characterized by expansion of lymphoid aggregates, T/B cell segregation, and lymphoid chemokine expression, which reaches a peak around day 15 p.c. and undergoes resolution with complete lymphocyte clearance by day 30 p.c. (Supplemental Fig. 1A). These aggregates are characterized by expansion of a gp38^+^/podoplanin^+^ fibroblast network, previously observed in human TLS (Supplemental Fig. 1B) (21).

**FIGURE 1.**
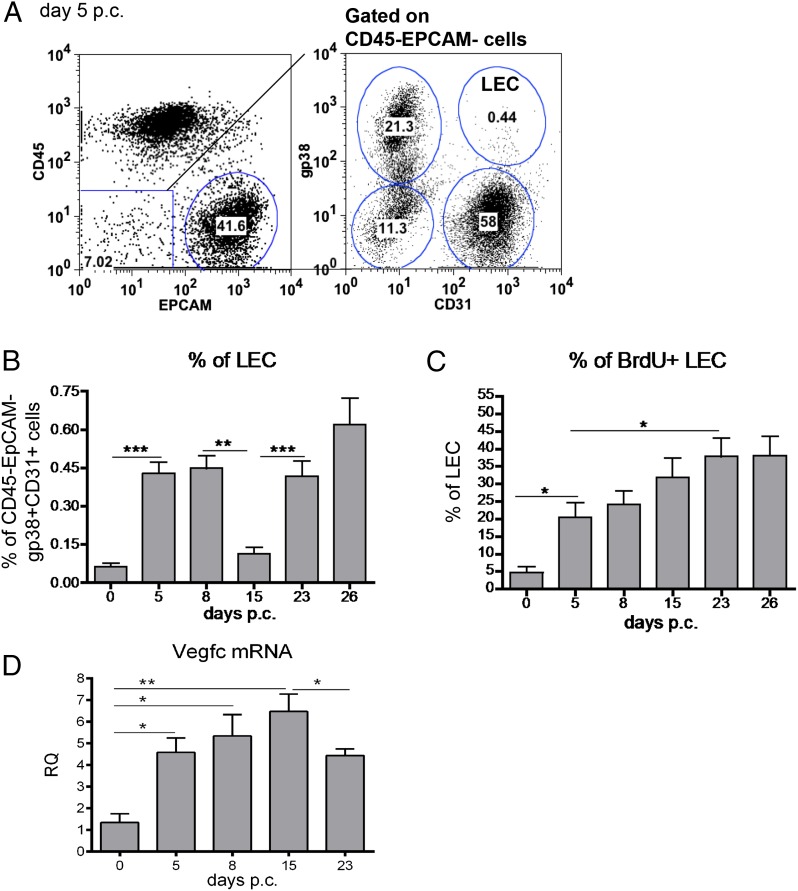
Bimodal expansion of the lymphatic bed during TLS development. (**A**) Representative dot plots showing flow cytometry staining for gp38 and CD31 in the CD45^−^EpCAM^−^ cells from salivary glands isolated at day 5 after viral cannulation. The LECs are identified as gp38^+^CD31^+^ cells. (**B**) Time course of LEC expansion during the inflammatory process determined by flow cytometry (percentage of gp38^+^CD31^+^ population in the CD45^−^EpCAM^−^ component) from infected wt mice at days 0, 5, 8, 15, 23, and 26 p.c. Data are presented as means of five independent experiments. **p < 0.01, ***p < 0.001, unpaired *t* test, comparing LEC population at each time point with day 0 p.c. LEC. (**C**) Graphs showing summary of analysis for percentage of proliferating (BrdU^+^) gp38^+^CD31^+^ LECs in the CD45^−^EpCAM^−^ stromal fraction. BrdU was administered from day 0 continuously. *p < 0.05, **p < 0.01 versus day 0 p.c. for wt mice. (**D**) Quantitative RT-PCR analysis of mRNA transcript for Vegfc in wt mice at days 0, 5, 8, 15, and 23 p.c. Transcripts were normalized to housekeeping gene β-actin. The RQ expression values were calibrated with day 0 p.c. salivary gland values. Data are representative of three to four independent experiments with six to eight glands analyzed per group. Data are shown as mean ± SEM. *p < 0.05, **p < 0.01.

Salivary glands of wild-type (wt) mice were cannulated with replication deficient adenovirus or vehicle control. Mice were sacrificed at specific time points p.c. and salivary glands were isolated as described (28). Flow cytometry on digested single-cell suspensions to evaluate the expansion of LECs within the EPCAM^−^CD45^−^ compartment (Fig. 1A) revealed a bimodal pattern of expansion of the gp38^+^CD31^+^ LECs. The first peak coincided with the initial establishment of the TLS and was observed around day 5–8 p.c. (Fig. 1B). A second phase of expansion occurred after day 15 p.c. and coincided with the beginning of the involution of the inflammatory foci (Fig. 1B, Supplemental Fig. 1B). Investigation of the proliferative status of the LECs, using BrdU incorporation, administered to the mice continuously from the day of the cannulation, revealed stable proliferation of this compartment between day 5 and day 23 p.c. (Fig. 1C). The proliferation observed in the LEC associates with a stepwise increase in vegfc transcript upon cannulation of the salivary glands (Fig. 1D).

### Remodeling of the lymphatic vascular network during TLS formation

**FIGURE 2.**
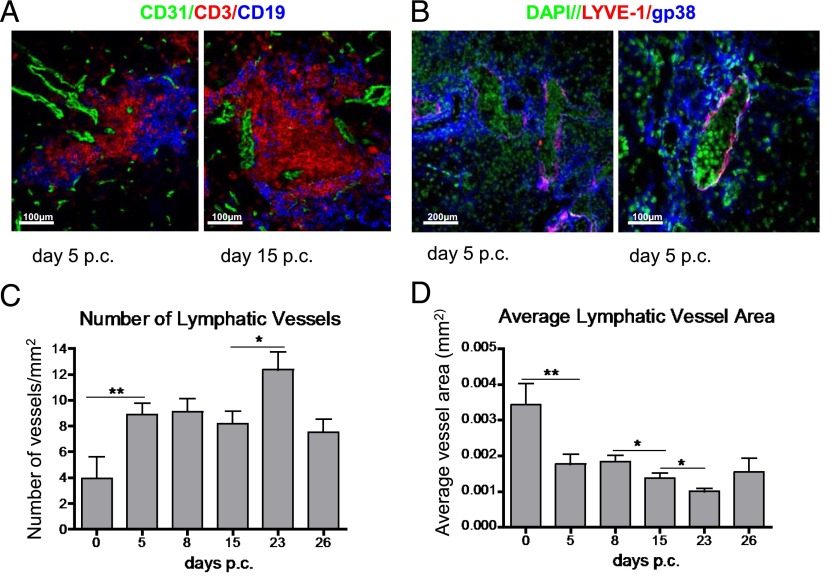
Remodeling of the lymphatic network during TLS development. (**A**) Photomicrograph of lymphoid aggregates from infected salivary glands (days 5 and 15 p.c.) from wt mice stained for CD3 (red), CD19 (blue), and CD31 (green). Original magnification, ×25. (**B**) Photomicrograph of lymphatic vessels in infected salivary glands (day 5 p.c.) from wt mice stained for LYVE-1 (red), gp38 (blue), and DAPI (green). Original magnification, ×10 and ×25. (**C** and **D**) Graphs summarizing image analysis in salivary gland tissue sections at different time points of the inflammatory process to identify changes observed in lymphatic vessel expansion. The graphs show the number of lymphatic vessels/mm^2^ of tissue area (C) and average vessel area expressed in mm^2^ (D). Data are representative of three to four independent experiments with four to six glands analyzed per group. Data shown as mean ± SEM. *p < 0.05, **p < 0.01 versus day 0 p.c. for wt mice.

Immunofluorescence analysis performed on dissected salivary glands after cannulation allowed direct visualization and quantification of the lymphatic network in the context of the lymphoid aggregates. A combination of CD31 and LYVE-1 staining showed the presence of lymphatic vessels in the glands with a tendency to localize in the outer part of the follicular aggregates at the earliest disease phases of TLS assembly (Fig. 2A, 2B). At this stage most lymphatic vessels were characterized by enlargement of the vascular lumen and the presence of lymphocytes within the vessels (Fig. 2B). This phenomenon was less evident in the later phases of the disease (day 23 p.c.) that were characterized by lymphatic vessels of smaller caliber, essentially devoid of lymphocytes (Supplemental Fig. 2).

Quantification of the area covered by the lymphatic network was achieved by using ImageJ analysis on whole-section tile scans (see *Materials and Methods*). A significant increase in the total number of vessels per tissue area was observed in the early phases of the TLS formation (day 5 p.c.) as compared with resting conditions. This increased number of vessels remained stable over time, with a further significant increase observed at day 23 p.c. (Fig. 2C). The average vessel area measured showed that an increase in lymphatic vessel number was associated with a reduction in the mean vessel lumen area, suggesting progressive splitting of the pre-existing vessels (Fig. 2D).

### Lack of LTα_1_β_2_ affects lymphangiogenesis in TLS

**FIGURE 3.**
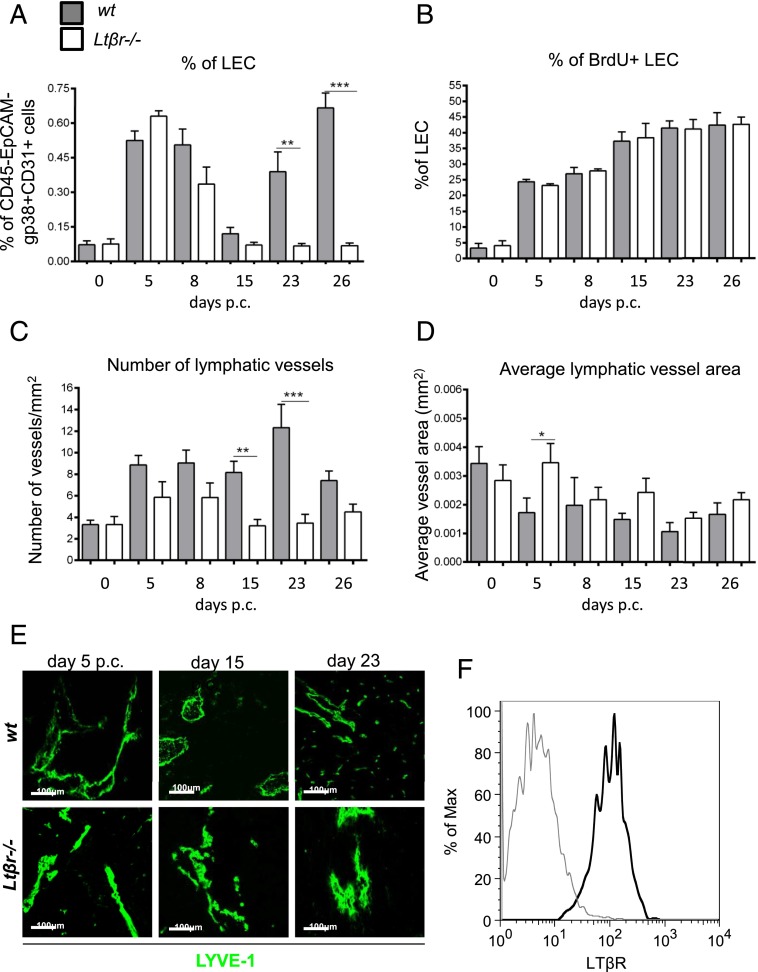
Lack of LTβ affects lymphangiogenesis in TLS. (**A**) Graph showing flow cytometry analysis of LEC expansion in wt mice (filled bars) compared with *LtβR^−/−^* (open bars) mice. *p < 0.05, **p < 0.01, unpaired *t* test, comparing gp38^+^CD31^+^ LEC population in infected knockout mice at various time points to their wt counterparts. (**B**) Graphs showing summary of analysis for percentage of proliferating (BrdU^+^) gp38^+^CD31^+^ LEC within the CD45^−^EpCAM^−^ stromal fraction in *wt* mice (filled bars) compared with *LtβR^−/−^* (open bars) mice. (**C** and **D**) Summarizing image analysis results showing differences observed in the number of lymphatic vessels/mm^2^ of tissue area (C) and average vessel area (mm^2^) (D) in wt mice (filled bars) compared with *LtβR^−/−^* mice (open bars). Data are representative of three independent experiments with four to six glands analyzed per group. Data are shown as mean ± SEM. *p < 0.05, **p < 0.01, ***p < 0.001, unpaired *t* test, comparing LYVE-1^+^ vessels in infected knockout mice at various time points to their wt counterparts. (**E**) Representative photomicrograph of lymphatic vessels in infected salivary glands (days 5, 15, and day 23 p.c.) from *Ltβr^−/−^* mice in comparison with wt mice stained for LYVE-1 (green). Scale bars, 100 μm. (**F**) Histogram showing LTβR expression (black) and isotype control (gray) on LECs in salivary glands at day 5 p.c.

LTβR signaling has been reported to play a key role in physiological lymphoneogenesis during SLO development (29, 30). To investigate the effects of this pathway in TLS-associated lymphangiogenesis, we cannulated *Ltßr* knockout mice (*Ltßr^−/−^*). Aggregates formed in *Ltßr^−/−^* mice but were characterized by reduced organization and diminished chemokine expression (data not shown). Accordingly, the dynamics of vascular expansion was altered in these animals. The first peak of LEC expansion in *Ltßr^−/−^* mice was similar to their wt counterparts. Conversely, from day 8 p.c. we observed a decrease in the percentage of the LECs in the *Ltßr^−/−^* that became significant at days 23 and 26 p.c. (Fig. 3A). Despite this defective expansion of the lymphatic network, the LECs from *Ltßr^−/−^* mice appeared to proliferate at the same rate compared with wt mice (Fig. 3B).

Image analysis of the cannulated salivary glands of *Ltßr^−/−^* mice demonstrated in the knockout mice a decrease in the number of the lymphatic vessels that reached significance at days 15 and 23 p.c. (Fig. 3C). This phenomenon was associated with a tendency in the *Ltßr^−/−^* mice to form lymphatic vessels with larger caliber as compared with the wt controls (Fig. 3D, 3E). Collectively, these data suggest that LTβR-mediated signals are involved in the induction of lymphangiogenesis within TLS. Indeed, expression of LTβR is detected on salivary gland LECs (Fig. 3F)

### The early phase of lymphatic vessel remodeling is dependent on IL-7

**FIGURE 4.**
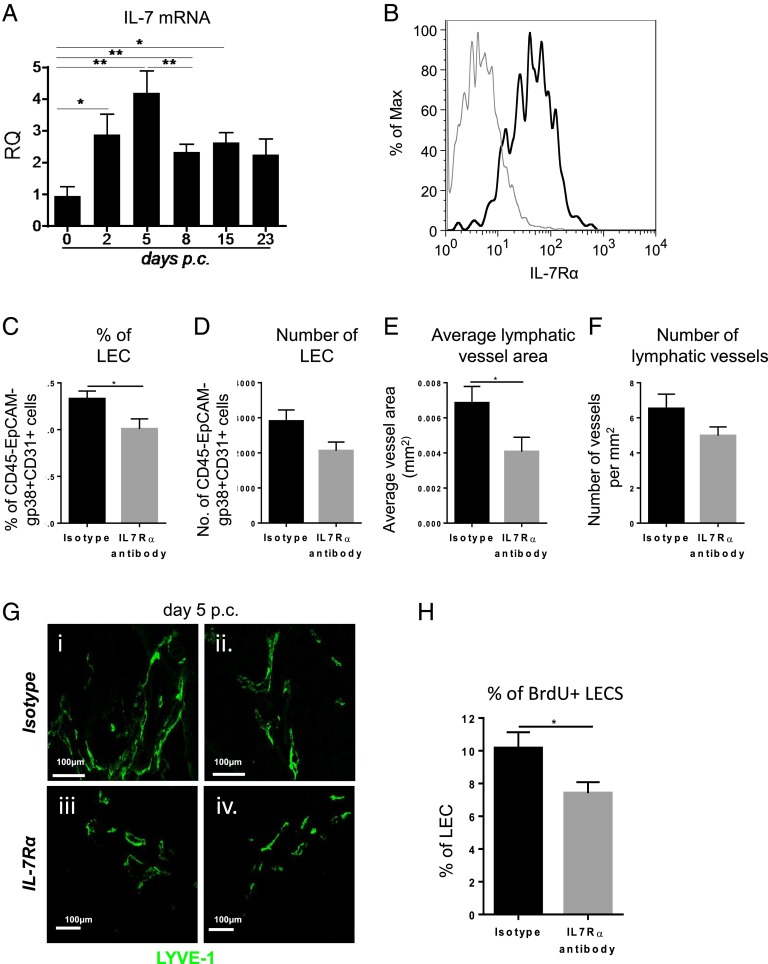
The early phase of lymphatic vessels remodeling is dependent on IL-7. (**A**) Quantitative RT-PCR analysis of mRNA transcript for il-7 in wt mice at days 0, 2, 5, 8, 15, and 23 p.c. Transcripts were normalized to housekeeping gene pdgfrß. The RQ expression values were calibrated with day 0 p.c. salivary gland values. Data are representative means ± SEM of three to four experiments with six to four glands analyzed per group. *p < 0.05, **p < 0.01. (**B**) Histogram showing IL-7Rα expression (black) and isotype control (gray) on LECs in salivary glands at day 5 p.c. (**C**) Graph showing flow cytometry analysis of percentage of LEC in wt mice treated with isotype Ab (black bars) as compared with IL-7Ra blocking Ab–treated mice (gray bars) mice. Data are represented as mean ± SEM. *p < 0.05. (**D**) Graph showing flow cytometry analysis of absolute number of LEC in wt mice treated with isotype Ab (black bars) as compared with IL-7Ra blocking Ab–treated mice (gray bars) mice. Data are represented as mean ± SEM. (**E** and **F**) Graphs summarizing image analysis results showing differences observed in the number of lymphatic vessels/mm^2^ of tissue area and average vessel area (mm^2^) in wt mice treated with IL-7Rα blocking Ab (gray bars) compared with isotype treated mice (black bars). Data are representative of two independent experiments with four to six glands analyzed per group. Data are shown as mean ± SEM. *p < 0.05, unpaired *t* test. (**G**) Representative photomicrograph of lymphatic vessels in infected salivary glands (day 5 p.c.) from IL-7Rα blocking Ab–treated mice (**i** and **ii**) in comparison with wt mice treated with isotype (**iii** and **iv**) stained for LYVE-1 (green). Scale bars, 100 μm. (**H**) Graphs showing summary of analysis for percentage of proliferating (BrdU^+^) gp38^+^CD31^+^ LEC within the CD45^−^EpCAM^−^ stromal fraction in wt mice treated with isotype Ab (black bars) as compared with IL-7Rα blocking Ab–treated mice (gray bars) mice. Data are represented as mean ± SEM. *p < 0.05.

Onder et al. (8) reported a critical role for IL-7 in lymphatic remodeling in SLOs. In our cannulation model, IL-7 expression was significantly increased and preceded the expansion of the lymphatic bed (Fig. 4A). Moreover, LECs specifically express IL-7Rα, thus displaying the machinery to respond to this homeostatic signal in vivo (Fig. 4B).

To investigate whether IL-7 was responsible for the first phase of expansion of the lymphatic bed, that is, intact in the *Ltßr^−/−^* mice, we treated wt-cannulated mice with a blocking Ab against IL-7Rα (see *Materials and Methods*). Treated mice display a significant decrease in LECs (Fig. 4C, 4D) and average lymphatic vessel area (Fig. 4E, 4G) and a smaller number of lymphatic vessels (Fig. 4F, 4G) that did not reach significance. This defect appeared to be due to a significant reduction in the proliferating ability of the LECs (Fig. 4H).

### LTα_1_β_2_ induces the formation of complex lymphatic networks

**FIGURE 5.**
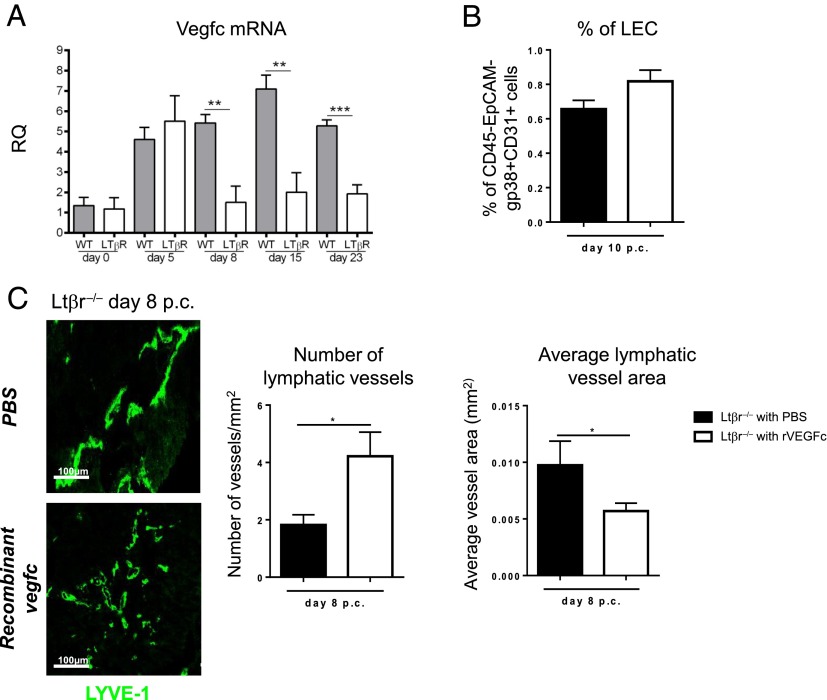
LTα_1_β_2_ induces the formation of complex lymphatic networks in vitro. (**A**) Quantitative RT-PCR analysis of mRNA transcript for Vegfc in *LtβR^−/−^* mice (open bars) in comparison with their wt counterparts (filled bars) at days 0, 5, 8, 15, and 23 p.c. Transcripts were normalized to housekeeping gene β-actin. The RQ expression values were calibrated with day 0 p.c. salivary gland values. *p < 0.05, **p < 0.01 versus wt mice. Data are representative of mean ± SEM of three to four experiments with six to four glands analyzed per group. (**B**) Graph showing flow cytometry analysis of percentage of LECs in *LtβR^−/−^* mice treated with recombinant VEGF-C (open bars) as compared with PBS-treated mice (filled bars) mice. Data are represented as mean ± SEM. (**C**) Representative photomicrograph of lymphatic vessels in infected salivary glands (day 8 p.c.) from recombinant vegfc-treated mice in comparison with PBS-treated *LtβR^−/−^* mice stained for LYVE-1 (green). Scale bars, 100 μm. Summarizing image analysis results show differences observed in the number of lymphatic vessels/mm^2^ of tissue area and average vessel area (mm^2^) in *LtβR^−/−^* mice treated with recombinant vegfc (open bars) as compared with PBS-treated mice (filled bars) mice. Data are representative of two independent experiments with four to six glands analyzed per group. Data are shown as mean ± SEM. *p < 0.05, unpaired *t* test.

Interestingly, VEGF-C mRNA transcripts were significantly decreased in *Ltßr^−/−^* mice (Fig. 5A), thus suggesting the possibility that the defect observed in the *Ltßr^−/−^* is sustained by a defect in VEGF-C induction. To test this hypothesis, we treated cannulated *Ltßr^−/−^* mice with recombinant VEGF-C (Fig. 5B) and observed a partial compensation of the defective phenotype described in these mice. VEGF-C–treated mice indeed displayed a significant increase in lymphatic vessels number accompanied by a decrease in the average vessel caliber (Fig. 5C), thus suggesting that although VEGF-C provides a positive signal to sustain vascular splitting, this does not completely explain the phenotype observed in the *LtβR^−/−^* mice.

**FIGURE 6.**
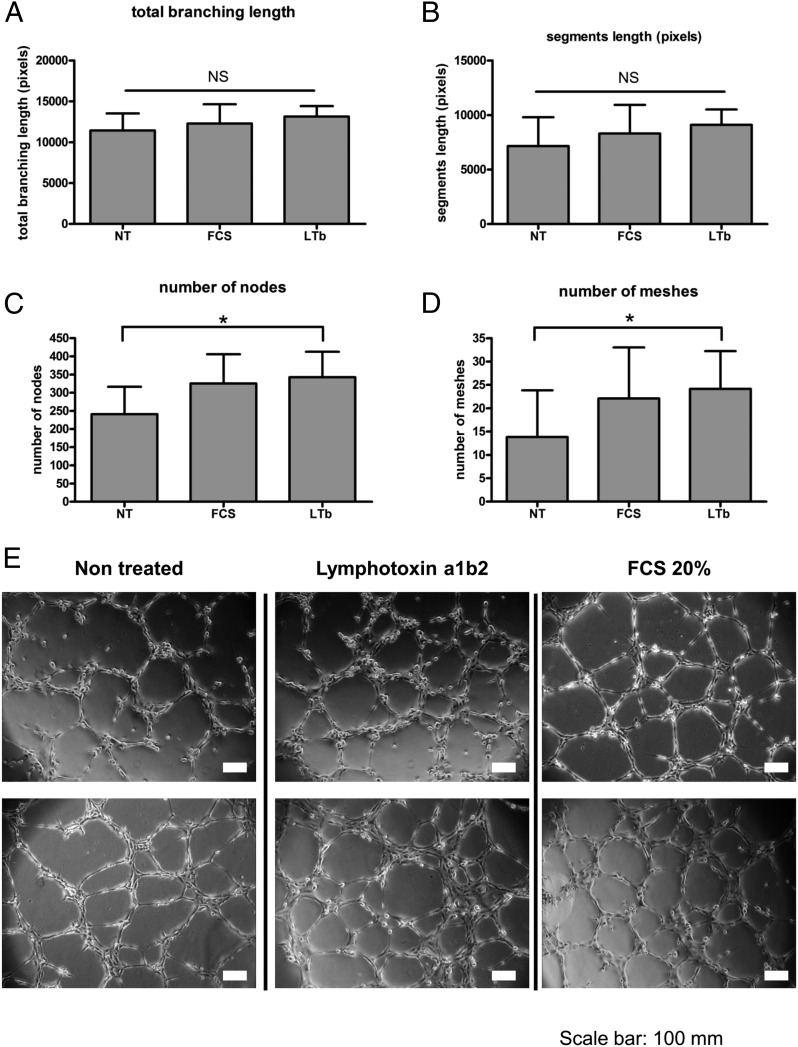
LTα_1_β_2_ induces the formation of complex lymphatic networks. In vitro analysis of the effect of LTα_1_β_2_ stimulation on lymphatic endothelial cell tube formation assay showing (**A**) total branching length, (**B**) segment length (pixels), (**C**) number of nodes, and (**D**) number of meshes. (**E**) Representative photomicrographs of nontreated, FCS-treated, and LTα_1_β_2_-treated lymphatic endothelial cells. Data are representative of three independent experiments. *p < 0.05 after one-way repeated measurements ANOVA analysis.

To evaluate whether LTα_1_β_2_ could alone influence TLS lymphangiogenesis, we used an in vitro tube formation assay in vitro. Primary human LECS were treated with either FCS or LTα_1_β_2_ (see *Materials and Methods*) and a series of parameters were collected upon imaging the cultures (Fig. 6). We observed no difference in proliferation, total branching length, or segments length of the tubes formed by the nontreated, FCS control–treated, and the LTα_1_β_2_-treated cells (Fig. 6A, 6B). On the contrary, we detected a significant increase both in the number of nodes (junctions) (Fig. 6C) and in the number of meshes (Fig. 6D, 6E) in the LTα_1_β_2_-treated cells, similar to that induced in FCS (serum)-treated positive control, as compared with nontreated samples. These data indicate that LTα_1_β_2_ facilitates the formation of a more sophisticated lymphatic network and are consistent with the observed phenotype of failed lymphangiogenesis present in the *Ltßr^−/−^* mice.

### Lymphatic vessel formation is influenced by the expression of LTα_1_β_2_ by Rorγ^+^ cells

**FIGURE 7.**
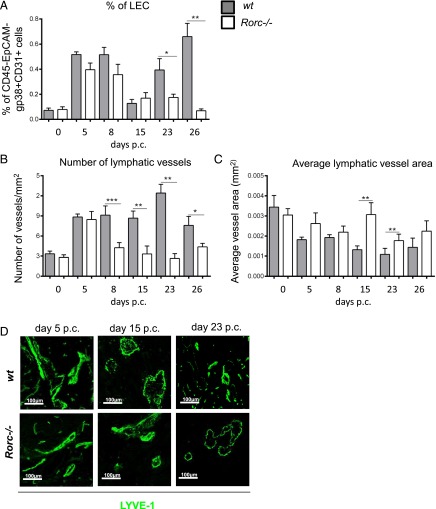
Lymphatic vessel formation is influenced by the expression of LTβ by Rorγ^+^ cells. (**A**) Graph showing flow cytometry analysis of LEC expansion in wt mice (filled bars) compared with *Rorc^−/−^* mice (open bars). Data are represented as mean ± SEM of two independent experiments. *p < 0.05, **p < 0.01, unpaired *t* test, comparing gp38^+^CD31^+^ LEC population in infected knockout mice at various time points to their wt counterparts. (**B**) Graphs showing the number of lymphatic vessels/mm^2^ of tissue in wt mice (filled bars) compared with *Rorc^−/−^* (open bars) mice. Data are representative of mean ± SEM of three independent experiments with four to six glands analyzed per group. *p < 0.05, **p < 0.01, ***p < 0.001, unpaired *t* test, comparing LYVE-1^+^ vessels in infected knockout mice at various time points to their wt counterparts. (**C**) Graphs showing average vessel area (mm^2^) in wt mice (filled bars) compared with *Rorc^−/−^* mice (open bars). Data are representative of mean ± SEM of three independent experiments with four to six glands analyzed per group. *p < 0.05, **p < 0.01, ***p < 0.001, unpaired *t* test, comparing LYVE-1^+^ vessels in infected knockout mice at various time points to their wt counterparts. (**D**) Representative photomicrograph of lymphatic vessels in infected salivary glands (days 5, 15, and day 23 p.c.) from *Rorc^−/−^* mice in comparison with wt mice stained for LYVE-1 (green). Scale bars, 100 μm.

It is known that Rorγt^+^ LTi cells represent the earliest source of LTα_1_β_2_ during physiological lymphoneogenesis (30, 31). To dissect the specific contribution of both these cellular components to TLS-associated lymphangiogenesis, we induced TLS formation in the salivary glands of *Rorc^−/−^* mice that are characterized by a defect in both LTi and Th17 cell formation. As predicted, the defect observed in the *Ltßr^−/−^* mice was largely reproduced in the *Rorc^−/−^* mice. In these mice we observed decreased LEC expansion as compared with wt mice (Fig. 7A). Moreover, we detected a significant increase in the average lymphatic vessel area and decreased number of vessels, suggesting a defect in lymphangiogenesis similar to that observed in the *Ltßr^−/−^* mice (Fig. 7B–D). This phenotype was not attributable to a proliferative defect, as shown by BrdU incorporation (Supplemental Fig. 3).

### Lack of lymphocytes affects both phases of lymphatic vessel expansion

**FIGURE 8.**
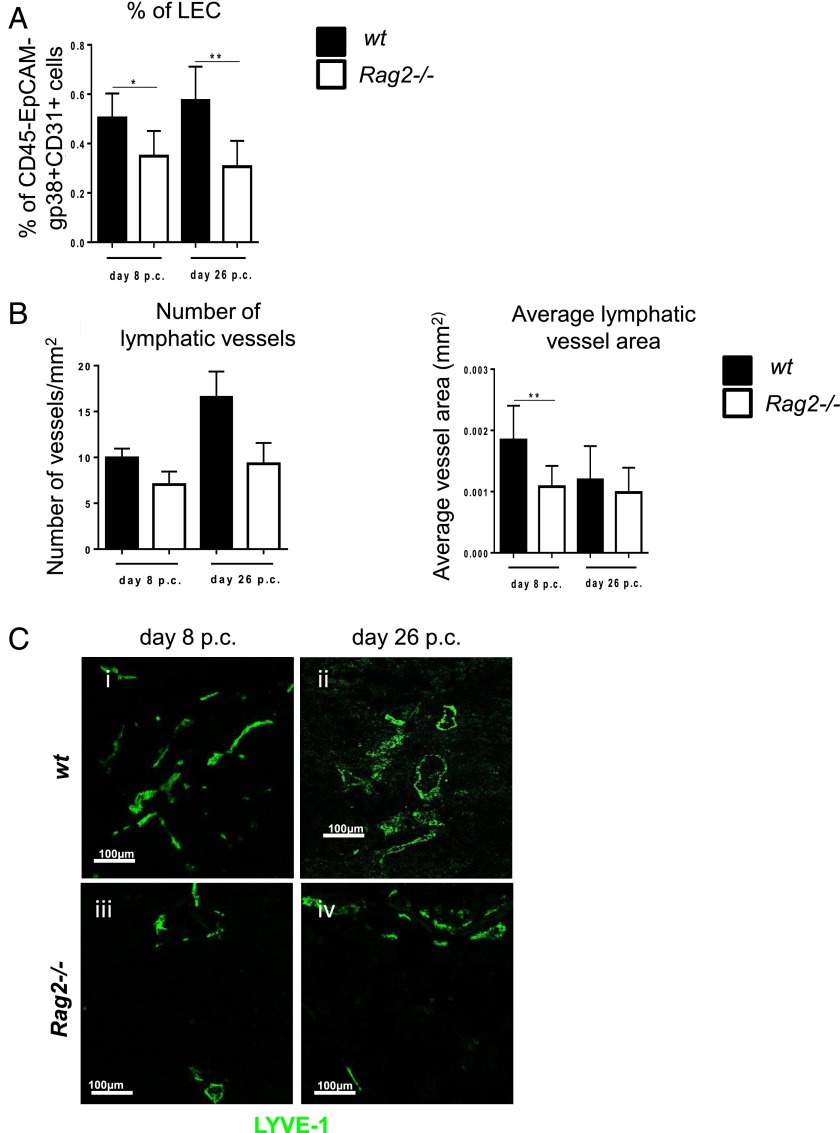
Lack of lymphocytes affects both phases of lymphatic vessel expansion. (**A**) Graph showing flow cytometry analysis of LEC expansion in wt mice (filled bars) compared with *Rag2^−/−^* mice (open bars) at days 8 and 26 p.c. Data are represented as mean ± SEM of two independent experiments. *p < 0.05, **p < 0.01, unpaired *t* test, comparing gp38^+^CD31^+^ LEC population in infected knockout mice at various time points to their wt counterparts. (**B**) Graphs showing number of lymphatic vessels and average vessel area (mm^2^) in wt mice (filled bars) compared with *Rag2^−/−^* mice (open bars). Data are representative of mean ± SEM of three independent experiments with four to six glands analyzed per group. **p < 0.01, unpaired *t* test, comparing LYVE-1^+^ vessels in infected knockout mice at various time points to their wt counterparts. (**C**) Representative photomicrograph of lymphatic vessels in infected salivary glands (days 8 and 26 p.c.) from *Rag2^−/−^* mice at day 8 p.c. (**iii**) and day 26 p.c. (**iv**) in comparison with wt mice at day 8 p.c. (**i**) and day 26 p.c. (**ii**) stained for LYVE-1 (green). Scale bars, 100 μm.

In adult SLOs, lymphocytes can compensate for the absence of LTi in the production of LTα_1_β_2._ Accordingly, cannulated *Rag2^−/−^* mice display a similar phenotype to the *Ltßr^−/−^* mice in the late phase of vascular expansion, accompanied by a significant defect in lymphangiogenesis even in the first wave of expansion, both in terms of LECs (Fig. 8A) and in the lymphatic vessel area and number, with the latter only showing a nonsignificant reduction (Fig. 8B, 8C). IL-7 transcripts in *Rag2^−/−^* mice showed a significant decrease as compared with wt mice (Supplemental Fig. 3).

## Discussion

During inflammation, expansion of the lymphatic vascular network is required to drain local edema, increase cellular output, and deliver infiltrating immune cells and Ags to draining lymph nodes. In order to accomplish these tasks lymphatic vessels undergo dramatic changes in shape and size that enable the clearance of immune cells and pathogens from the affected tissue (2).

TLS are aberrant accumulations of lymphocytes that form preferentially within inflamed mucosal sites and acquire features and functions similar to lymphoid tissue. In many diseases, TLS persistence is associated with worse disease outcome and, in some cases, development of lymphoid malignancies (15, 20, 32). In the lymph node, expansion of the lymphatic vascular network synchronizes with the enlargement of the lymphoid stroma and the increase in the influx of lymphocytes to preserve tissue homeostasis (2, 12). In TLS it is thought that this drainage system is defective and that lymphocyte accumulation occurs as a result of insufficient expansion of the lymphatic network. Indeed, failure to drain activated lymphocytes from the tissue might contribute to the persistence of tissue inflammation (33–35).

In this study, we examined the complex phenomenon of lymphoneogenesis, evaluating the expansion of LECs as well as the changes in shape and size of the lymphatic vessels by immunofluorescence. In this work we demonstrate that the increase in lymphatic vessels is initiated in the early phases of TLS development, but that this process is delayed and impaired when the aggregates reach full size and a higher degree of organization. At this stage lymphoid aggregates are surrounded by lymphocytes engulfed in lymphatic vessels, thus suggesting a reduction in the ability of the vascular network to drain the recruited lymphocytes. Interestingly, this phenomenon is reversed in the resolution phase of the TLS when a second wave of lymphatic vessel expansion occurs, together with the formation of small-caliber lymphatic vessels that contribute to tissue clearance.

In our model the expansion of the lymphatic vascular network is preceded by a significant increase in both IL-7 and LTα_1_β_2_ expression within the tissue, with both known to regulate leukocyte homeostasis and lymphangiogenesis in SLOs (36, 37) (Fig. 4A, Supplemental Fig. 4). IL-7 autocrine signal on the LECs in SLOs has been shown to regulate lymphatic vessel remodeling and expansion (9). Additionally, fibroblast-derived IL-7 appears to support lymphangiogenesis in a paracrine manner (8), thus suggesting IL-7 as key regulator of lymphatic vessel expansion in TLS. Indeed, prophylactic block with IL-7 affects LEC proliferation, determining the formation of lymphatic vessels with smaller caliber. Although this defect is not complete, it suggests a role for IL-7 in the early phases of TLS-associated lymphangiogenesis.

During ontogeny, LTβR triggering has been also shown to activate surrounding stromal cells to produce VEGF-C, a crucial mediator of lymphangiogenesis (11). In our system, genetic deletion of LTβR results in a reduction of lymphatic vessel number and size and a significant reduction in VEGF-C transcript expression after cannulation. Treatment of *LtβR^−/−^* mice with recombinant VEGF-C was only partially able to restore the *LtβR^−/−^* defect, suggesting a direct role for the latter in lymphatic vessel remodeling. Interestingly, blocking LTα_1_β_2_ did not influence LEC proliferation, as expected, but rather it decreased the complexity of the vascular network in the cannulated samples. Accordingly, it has been previously shown that blocking VEGF receptor signaling can inhibit formation of the complex tube of umbilical vein endothelial cells and LECs (38–40).

Previous studies have indicated a role for lymphocytes in lymphangiogenesis (2, 37, 41–43). In our model, the second phase of expansion of the lymphatic vessels is similarly affected by lack of LTα_1_β_2_ and Rorγt^+^ cells. We demonstrated that *Rorc^−/−^* mice, although forming normal TLS (data not shown), are characterized by a significant defect in lymphatic vessel expansion, similar to that observed in the *LtβR^−/−^*mice. This, associated with the defect in LTα_1_β_2_ observed in the *Rorc^−/−^* mice, suggests that Rorγt^+^ represent a critical source of LTα_1_β_2_ during TLS formation. Although the role of adult LTi in TLS formation is still debated, the role of Rorγt^+^ Th17 in TLS establishment and chemokine expression at ectopic sites is well recognized (44, 45). In this study, we suggest that Rorγt^+^ cells providing LTα_1_β_2_ play a larger and more complex role within TLS, regulating their homeostatic resolution in the tissue.

Interestingly, the defect observed in the lymphatic vessels in the absence of mature lymphocytes is already present in the early phases of TLS establishment, in a phase that is independent from LTβR activity. The decrease in IL-7 transcript observed in the *Rag2^−/−^* mice could justify this defective phenotype and deserves further investigation (Supplemental Fig. 3).

Lymphocyte-derived LTα_1_β_2_ is important for the full acquisition of lymphoid features by the TLS and has therefore been identified as a suitable target for TLS-associated disease (15, 46). Unfortunately, clinical trials that block the LT pathway in rheumatoid arthritis failed to meet primary end points, thus suggesting a more complex or redundant role for this molecule in the system (47). Our data support a critical role for LTα_1_β_2_ in TLS-associated lymphoneogenesis and provide a potential explanation as to why blocking the LT pathway in TLS-associated diseases may not be effective based on a requirement for the expansion of lymphatic vessels to enable lymphocyte egress during the resolution phase of inflammation.

## Supplementary Material

Data Supplement
