# Supplementary material for: Bimodal Expansion of the Lymphatic Vessels Is Regulated by the Sequential Expression of IL-7 and Lymphotoxin α1β2 in Newly Formed Tertiary Lymphoid Structures
Source: J Immunol. 2016 Jul 29;197(5):1957–67. doi: 10.4049/jimmunol.1500686 (PMC4991245; doi:10.4049/jimmunol.1500686)
Supplement: Data Supplement [file JI_1500686.zip › JI_1500686_Supplemental_Figures_1.pdf]

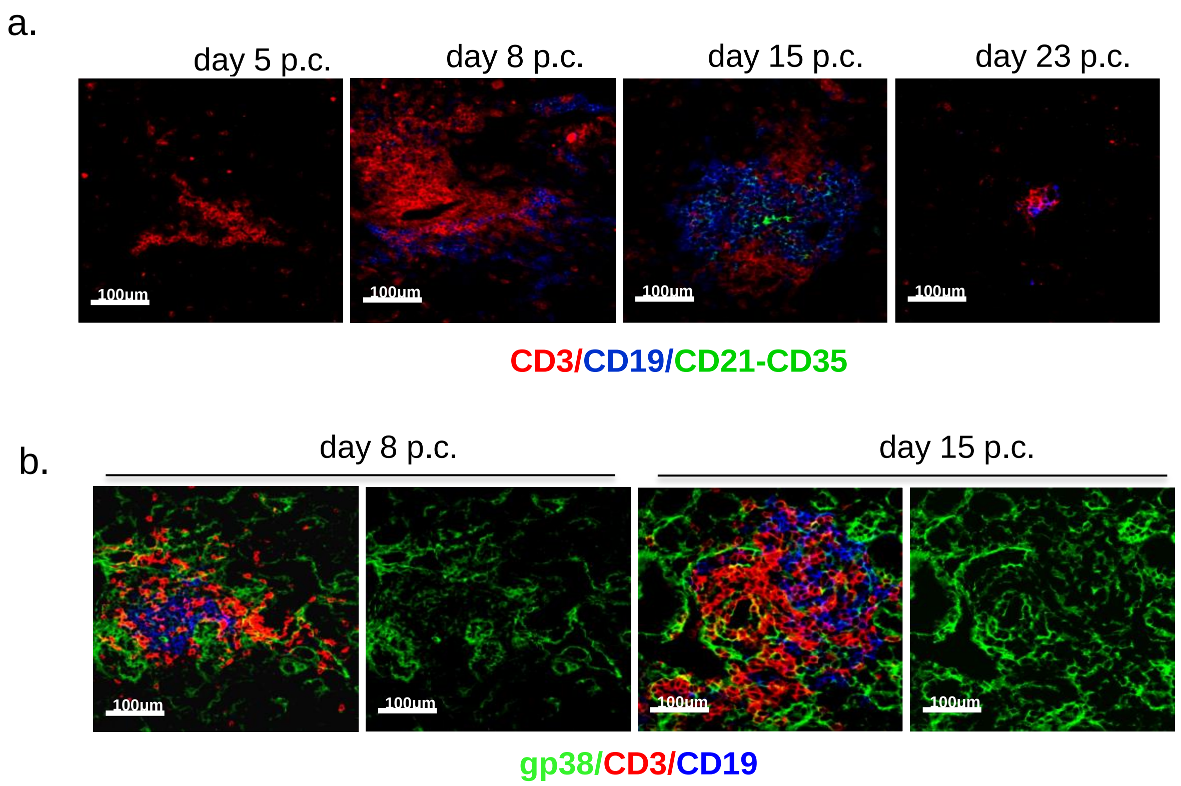

Supplementary Figure 1. Microphotographs of lymphoid aggregates in infected salivary glands (day 5, day 8, day 15 and day 23 p.c.) from *wt* mice stained for CD3 (red),CD19 (blue) and CD21-CD35 (green). Original magnifications 25X. **b,** Microphotograph of lymphoid stromal cell expansion within lymphoid aggregates in infected salivary glands (day 8 and 15 p.c.) from *wt* mice, stained for CD3 (red), CD19 (blue) and gp38 (green). Original magnifications 25X.

a. LYVE-1

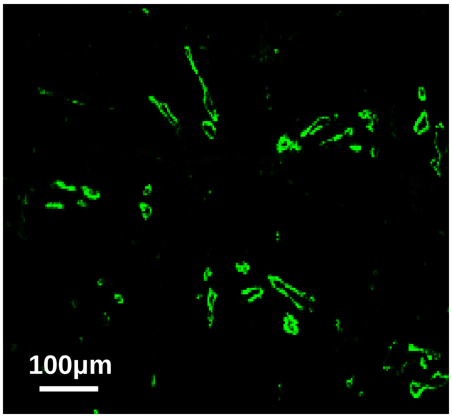

day 23 p.c.

Supplementary Figure 2. Representative microphotograph of lymphatic vessels in infected salivary glands (day 23 p.c.) from *wt* mice stained for LYVE-1 (green). Original magnifications 25X.

a.

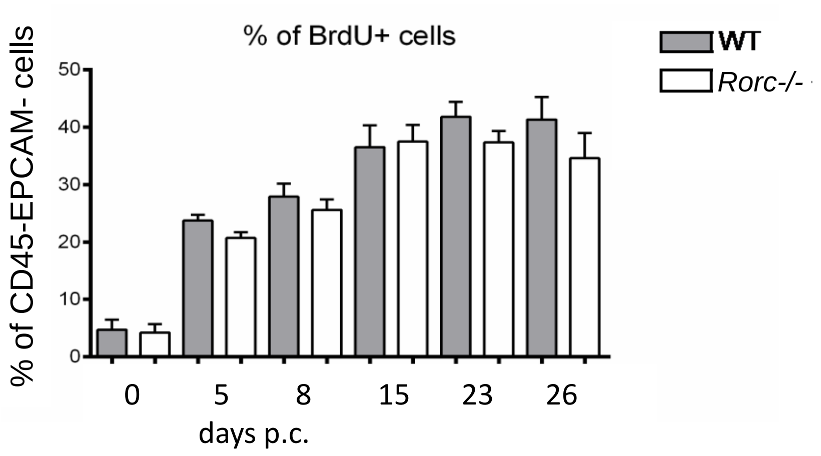

b.

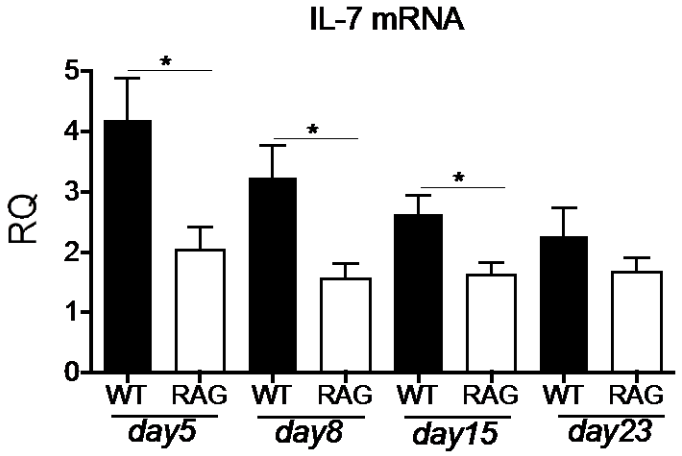

Supplementary Figure 3 a, Graphs showing summary of analysis for percentage of proliferating (BrdU+) gp38+CD31+ LEC within the CD45-EPCAM- stromal fraction in *wt* mice (grey bars) compared to *Rorc-/-* mice (white bars). Data represented as mean+s.e.m of two independent experiments. **b**, Quantitative RT-PCR analysis of mRNA transcript for IL-7 in *wtboyJ* and ragboyJ mice at day 5, 8, 15 and 23 p.c. Transcripts were normalized to housekeeping gene pdgfrβ. The relative expression values (RQ) were calibrated with day 0 p.c. salivary gland values; $*$, $p < 0.05$. Data are representative mean+s.e.m of three to four experiments with six to four glands analysed per group.

a.

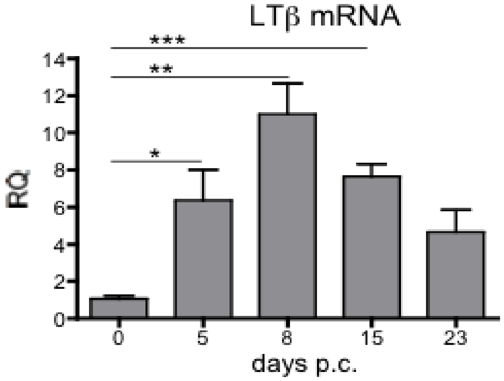

Supplementary Figure 4, Quantitative RT-PCR analysis of mRNA transcript for Ltβ in *wt* mice at day 0, day 5, 8, 15 and 23 p.c. Transcripts were normalized to housekeeping gene β-actin. The relative expression values (RQ) were calibrated with day 0 p.c. salivary gland values; ∗, $p < 0.05$, ∗∗, $p < 0.01$, ∗∗∗, $p < 0.001$. Data are representative mean+s.e.m of three to four experiments with six to four glands analysed per group.
